# Hyperglycemia and advanced glycation end products (AGEs) suppress the differentiation of 3T3-L1 preadipocytes

**DOI:** 10.18632/oncotarget.18993

**Published:** 2017-07-05

**Authors:** Chia-Chu Chang, Chen-Yu Chen, Geen-Dong Chang, Ting-Huan Chen, Woan-Ling Chen, Hui-Chin Wen, Chih-Yang Huang, Chung-Ho Chang

**Affiliations:** ^1^ Graduate Institute of Basic Medical Science, Ph.D. Program for Aging, China Medical University, Taichung, Taiwan 40402, Republic of China; ^2^ School of Medicine, Chung Shan Medical University, Taichung, Taiwan 40201, Republic of China; ^3^ Environmental and Precision Medicine Laboratory, Department of Internal Medicine, Changhua Christian Hospital, Changhua, Taiwan 50006, Republic of China; ^4^ Institute of Cellular and System Medicine, National Health Research Institutes, Zhunan, Miaoli, Taiwan 35053, Republic of China; ^5^ Graduate Institute of Biochemical Sciences, School of Life Science, National Taiwan University, Taipei, Taiwan 10617, Republic of China; ^6^ Department of Life Science, National Tsing Hua University, Hsinchu, Taiwan 30013, Republic of China; ^7^ Department of Food Science, Tunghai University, Taichung, Taiwan 40704, Republic of China

**Keywords:** advanced glycation end products (AGEs), hyperglycemia, adipogenesis, NF-kB, Bcl-2

## Abstract

Aging is characterized by mild hyperglycemia and accumulation of advanced glycation end products (AGEs). Effects of chronic exposure to hyperglycemia or AGEs on the adipogenic differentiation of 3T3-L1 preadipocytes remain unclear. We examined the chronic effect of AGEs and high glucose on the differentiation of 3T3-L1 cells by culturing 3T3-L1 cells in the presence of AGEs or 25 mM glucose for 1 month. Chronic incubation of 3T3-L1 cells with AGEs or high glucose blocked their differentiation into mature adipocytes as evidenced by reduced levels of adipocyte markers such as accumulated oil droplets, GPDH, aP2, adiponectin and of adipogenesis regulators PPARγ and C/EBPα. Levels or activities of Src, PDK1, Akt, and NF-κB were higher in AGEs- and high glucose-treated cells than those in 3T3-L1 cells. Levels of Bcl-2 were elevated in AGEs- and high glucose-treated cells, and were attenuated by inhibitors of PI3-kinase, Akt and NF-κB. Moreover, adipogenesis was attenuated in 3T3-L1 cells stably expressing Bcl-2 or YAP. These results suggest that chronic AGEs and high glucose treatments up-regulate Bcl-2 and YAP via the Akt-NF-κB pathway and impair adipogenesis.

## INTRODUCTION

Aging is known to attenuate the differentiating ability of preadipocytes into adipocytes [1–3]. Expression of transcriptional factors that regulate adipogenesis declines with aging in rat preadipocytes from various ages. However, the underlying mechanisms remain unclear. Advanced glycation end products (AGEs) are formed *in vivo* by a non-enzymatic reaction of proteins, lipids and nucleic acids, with glucose and other reducing sugars [4–7]. AGEs accumulation occurs in many tissues during aging due to a moderate increase in fasting glucose and to long-term exposure of proteins to normoglycemic condition [8–10]. Therefore, AGEs and mild hyperglycemia may mediate some of the inhibitory effects of aging on adipogenesis.

Effects of AGEs and high glucose on the differentiation of progenitor cells or preadipocytes are not well understood. High glucose has been shown to induce the differentiation of muscle-derived stem cells into adipocytes [11] and promote adipogenic differentiation of bone marrow-derived mesenchymal stem cells [12]. In the case of 3T3-L1 preadipocytes, 25 mM glucose was reported to inhibit adipogenesis [13]. In contrast, Lin *et al*. showed that there is no difference in the adipogenic differentiation of 3T3-L1 preadipocytes in the presence of 25 mM or 4 mM glucose [14]. On the other hand, AGEs have been shown to attenuate the differentiation of 3T3-L1 cells and human mesenchymal stem cells into adipocytes [15, 16]. In contrast, we and others have shown that short-term AGEs stimulation promoted the differentiation of 3T3-L1 preadipocytes [17, 18]. However, in these previous studies, cultured cells were only exposed to high glucose or AGEs in the early stage of differentiation or during the whole differentiation process (*i.e*., less than 7 days). Since adipocytes and preadipocytes turnover slowly [19], the chronic effects of AGEs and hyperglycemia on adipogenesis and the underlying mechanisms remain to be established.

To mimic the conditions of aging, we cultured 3T3-L1 cells in the presence of AGEs or 25 mM glucose for 1 month, and then examined the effects of these chronic treatments on the differentiating ability of 3T3-L1 preadipocytes. Our results showed that the ability of AGEs- and high glucose-treated 3T3-L1 cells to differentiate into mature adipocytes was impaired as these cells expressed low levels of adipocyte markers such as oil droplets, GPDH, aP2 and adiponectin and reduced levels of PPARγ and C/EBPα. Our studies further indicated that the Src kinase-Akt and PI3-K-PDK1-Akt-NF-κB pathways were elevated, and that levels of NF-κB genes, Bcl-2 and YAP, were up-regulated in AGEs- and high glucose-treated 3T3-L1 cells. Adipogenesis was also impaired in 3T3-L1 cells overexpressing Bcl-2 or YAP. Thus, chronic AGEs or high glucose treatment inhibits the differentiation of 3T3-L1 preadipocytes at least partly by elevating Bcl-2 and YAP.

## RESULTS

### Blockage of adipogenic differentiation of 3T3-L1 preadipocytes by hyperglycemia and AGEs

**Figure 1:**
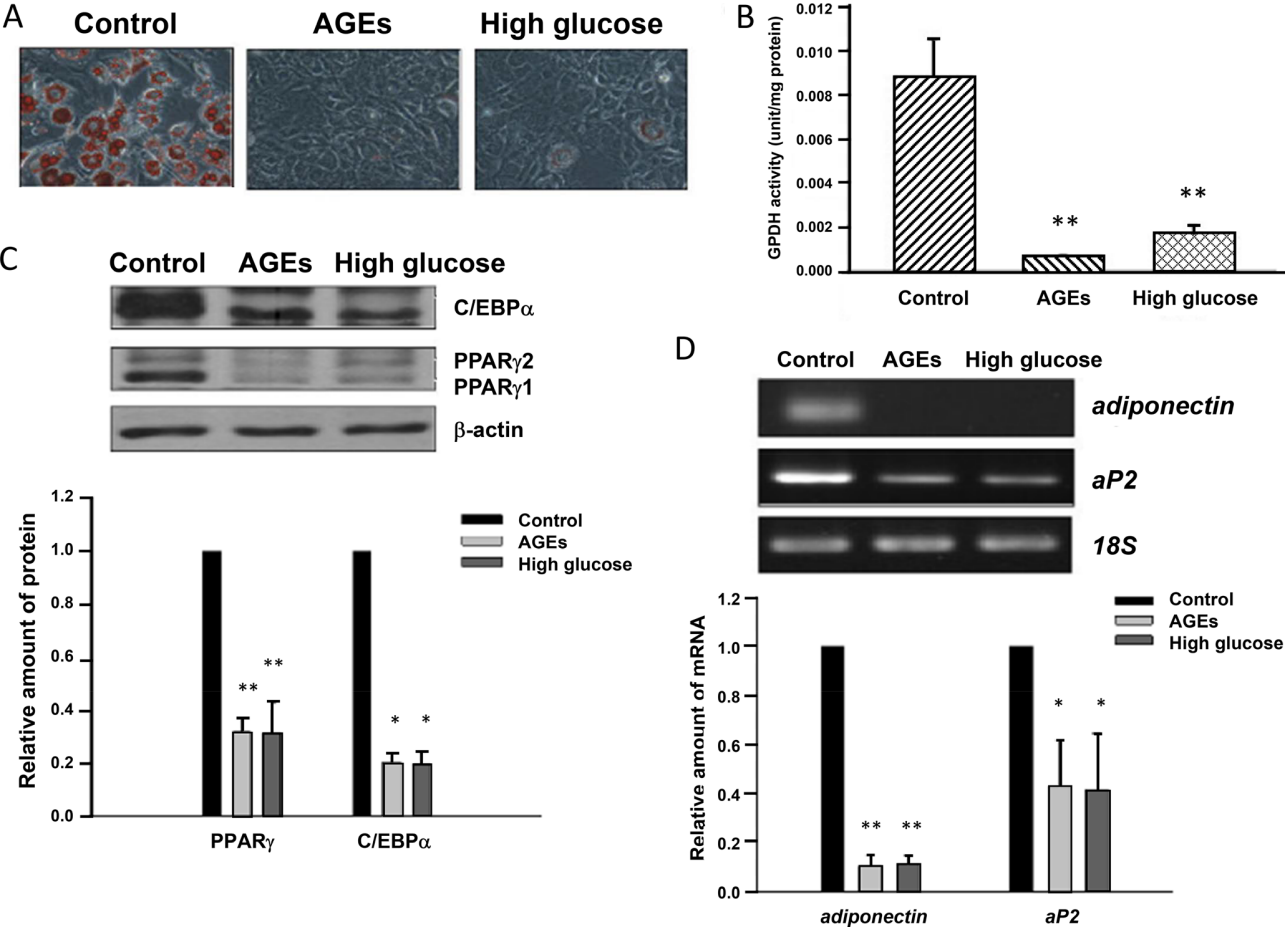
Blockage of 3T3-L1 preadipocyte differentiation by AGEs and high glucose (**A**) 3T3-L1, AGEs- and 25 mM glucose-treated preadipocytes were induced to differentiate into adipocytes. Eight days after induction, lipid droplets were visualized by the Oil red O staining. Representative microscope images were shown. (**B**) 3T3-L1, AGEs- and high glucose-treated cells were harvested on day 8 after adipogenic induction and subjected to GPDH activity assay. The results are indicated as the means ± SEM of three independent experiments. (**C**) Seven days after induction into adipocytes, cell lysates from control, AGEs- and high glucose-treated cells were immunoblotted with C/EBPα, PPARγ and β-actin antibodies. Bands were quantified by densitometry analysis, and values are the means ± SEM of three independent experiments. (**D**) On day 9 after differentiation induction, total RNAs of control, AGEs- and high glucose-treated 3T3-L1 adipocytes were isolated. The semi-quantitative RT-PCR was used to examine the expression levels of adiponectin, aP2 and 18S ribosomal RNA. Data were representative of three independent experiments. Symbols * and ** indicate the statistically significant differences when *p* < 0.05 and *p* < 0.01 as compared to control, respectively.

Adipose tissues compose of adipocytes, preadipocytes and other cell types. Adipocytes and preadipocytes are slow turnover cells among these diversified cell types [19]. To mimic the condition of aging, we cultured 3T3-L1 cells in the presence of 25 mM glucose or 900 μg/mL AGEs for one month. The differentiation inducing agents including 1.7 μM insulin, 0.5 mM IBMX and 1 μM dexamethasone were added to 3T3-L1, AGEs- and high glucose-treated cells. Oil red O (ORO) was used to stain the oil droplets within adipocytes. Figure 1A showed that 3T3-L1 cells differentiated into adipocytes in 8 days. Interestingly, unlike 3T3-L1 cells, the differentiation of glucose- and AGEs-treated cells was blocked (Figure 1A). Since GPDH is another widely used marker for adipocytes, GPDH activities of these 3T3-L1 cells were assessed [20]. GPDH activities were lower in AGEs- and high glucose-treated 3T3-L1 cells than that in 3T3-L1 cells. We have also treated 3T3-L1 cells with 100 μg/mL AGEs for one month and obtained similar results (as shown in Supplementary Figure 1). These results indicate that AGEs and high glucose treatments inhibit adipogenesis of 3T3-L1 preadipocytes (Figure 1B).

### Altered expression levels of adipocyte-specific genes by chronic hyperglycemia and AGEs treatments

Chronic treatments with high glucose and AGEs may alter the expression of genes regulating the preadipocyte differentiation. We examined the expression of several adipocyte-specific markers or transcription factors during the differentiation of untreated, high glucose- and AGEs-treated 3T3-L1 cells. Figure 1C showed that 7 days after induction, levels of PPARγ and C/EBPα were much lower in high glucose- and AGEs-treated cells than those in untreated 3T3-L1 cells. In contrast, there were little differences in the expression levels of C/EBPβ and C/EBPδ in differentiating 3T3-L1, high glucose- and AGEs-treated cells on day 1 (as shown in Supplementary Figure 2). Consistently, on day 9 after differentiation induction, levels of PPARγ-regulated genes aP2 and adiponectin, markers for adipocytes, in glucose- and AGEs-treated 3T3-L1 cells were also lower than those in control cells (Figure 1D).

### Elevated activation of Src and PI3-kinase-Akt in high glucose- and AGEs-treated 3T3-L1 cells

**Figure 2:**
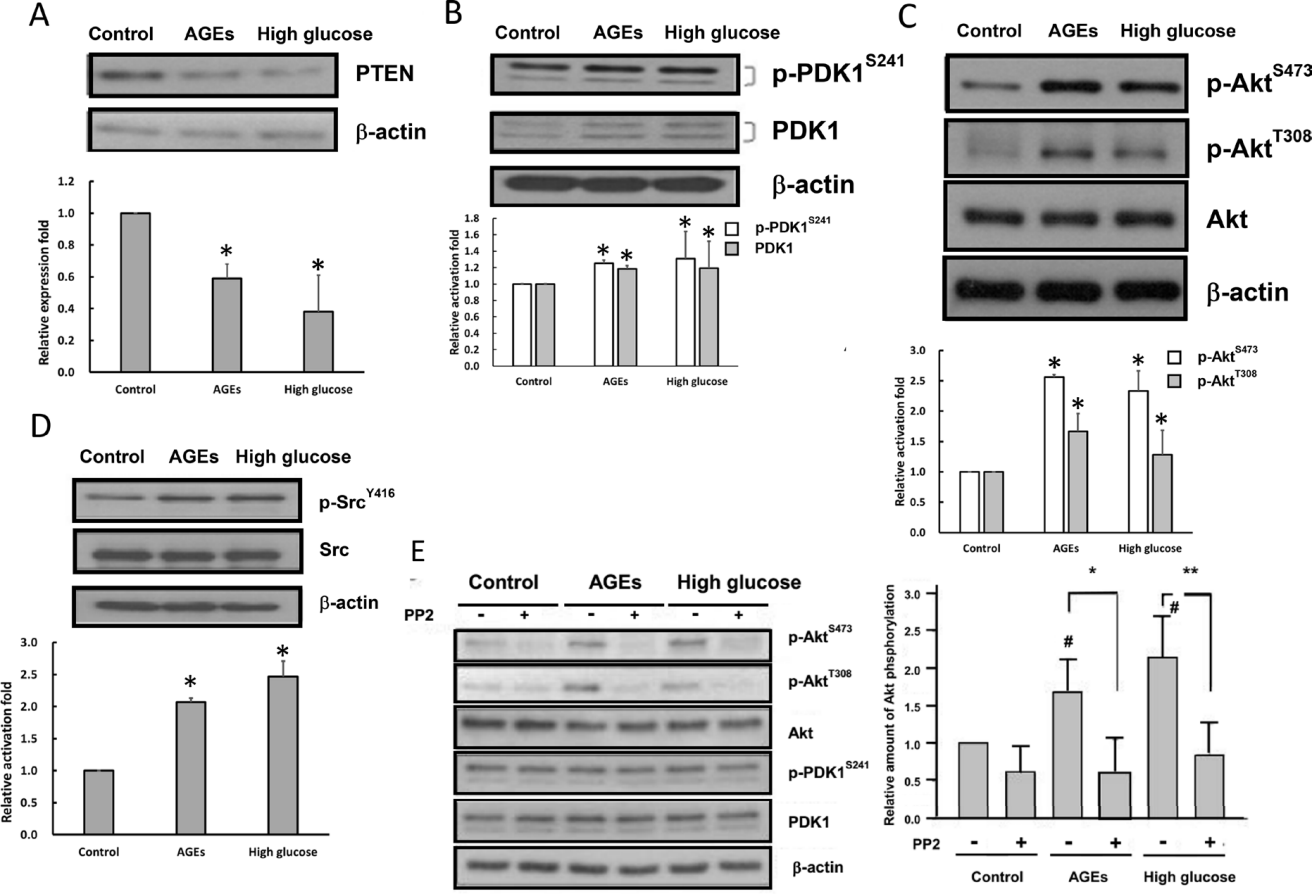
Src kinase and PI3-kinase-Akt pathway are activated in high glucose- and AGEs-treated 3T3-L1 cells Cell lysates from control, AGEs- and high glucose-treated cells were prepared and immunoblotted with PTEN (**A**), PDK1, p-PDK1 ^S241^ (**B**), Akt, p-Akt ^S473^, p-Akt ^T308^ (**C**), Src, p-Src ^Y416^ (**D**), or β-actin antibodies. (**E**) Control, AGEs- and high glucose-treated cells were serum-deprived overnight and treated with 10 μM PP2 for 30 minutes. Cell lysates were subjected to Western blot analysis using indicated antibodies. The values listed at the bottom of each lane indicate the relative changes normalized to control. Bands were quantified by densitometry analysis, and values shown are the means ± SEM of three independent experiments. # indicates the statistical significance between treatment and control groups. Symbols * and ** indicate the statistical significances between treatments with and without 10 μM PP2 as *p* < 0.05 and *p* < 0.01, respectively.

The PI3-kinase-PDK1-Akt pathway is involved in the regulation of many physiological processes. We examined whether the PI3-kinase-PDK1-Akt pathway is altered in high glucose- and AGEs-treated cells. Figure 2A showed that protein levels of PTEN were decreased in high glucose- and AGEs-treated 3T3-L1 preadipocytes. PDK1 protein levels and activation of PDK1 and Akt were enhanced in high glucose- and AGEs-treated 3T3-L1 cells as compared to those in control cells (Figure 2B and 2C).

Src has been shown to activate the PI3-kinase-PDK1-Akt pathway [21–23]. Western blotting with the phospho-Y416-Src antibody was performed to examine whether Src is activated in AGE- and high glucose-treated 3T3-L1 cells. Figure 2D showed that the phosphorylation levels of Y416 on Src were enhanced in AGE- and high glucose-treated 3T3-L1 cells as compared to those in control cells. To examine whether Src is upstream of the PI3-kinase-Akt pathway, we measured the effects of PP2 that is a Src inhibitor on PDK1 and Akt activities in 3T3-L1, AGEs- and high glucose-treated cells. Figure 2E showed that addition of PP2 inhibited Akt, but not PDK1, activity in all three cell types. Therefore, chronic AGEs and high glucose treatments may activate Akt via both Src-dependent and PI3-kinase/PDK1-dependent pathways.

### Chronic AGEs and high glucose treatments up-regulate Bcl-2 via Akt and NF-κB

**Figure 3:**
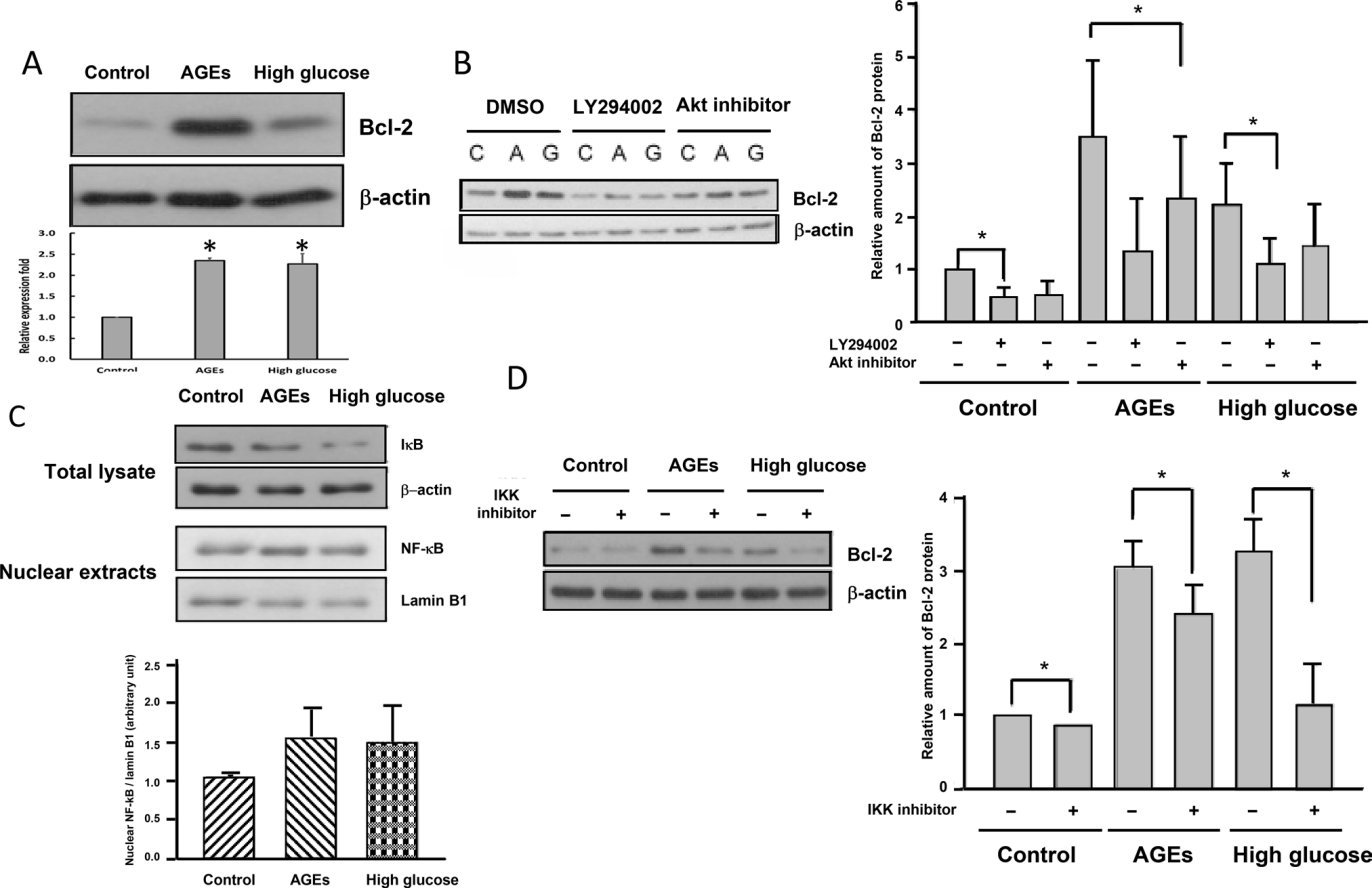
Elevated Akt and NF-kB activation lead to up-regulation of Bcl-2 in AGEs- and high glucose-treated cells (**A**) Cell lysates from control, AGEs- and 25 mM glucose-treated cells were immunoblotted with Bcl-2 or β-actin antibodies. Control, AGEs- and high glucose-treated cells were treated with 15 μM LY294002 or 10 μM Akt inhibitor (**B**) or 1 μM IKK inhibitor (**D**) for 48 hours before harvest. Cell lysates were subjected to Western blot analysis with Bcl-2 or β-actin antibodies. (**C**) Total cell lysates and nuclear extracts from control, AGEs- and high glucose-treated cells were subjected to Western blot analysis using IκB, NF-κB, laminB1 or β-actin antibodies. Bands were quantified by densitometry analysis, and values shown in the figures are the means ± SEM of three independent experiments. *p < 0.05, as compared to control.

Akt is known to up-regulate Bcl-2 in a variety of cell types [24, 25]. Western blot analysis revealed that Bcl-2 levels in AGEs- and high glucose-treated cells were much higher than those in 3T3-L1 cells (Figure 3A). Addition of LY294002 or Akt inhibitor attenuated levels of Bcl-2 (Figure 3B) in AGEs- and high glucose-treated cells, indicating that elevated Akt activation leads to up-regulation of Bcl-2 in AGEs- and high glucose-treated cells.

Bcl-2 is a known target gene of NF-κB [26], which is activated by Akt [27, 28]. Figure 3C showed that IκB levels were reduced in AGEs- and high glucose-treated cells, whereas nuclear NF-κB levels were higher in AGEs- and high glucose-treated cells than 3T3-L1 cells, suggesting that NF-κB was activated in AGEs- and high glucose-treated cells as compared to 3T3-L1 cells. Addition of IKK inhibitor attenuated levels of Bcl-2 (Figure 3D) in AGEs- and high glucose-treated cells, indicating that elevated Akt and NF-κB activation leads to up-regulation of Bcl-2 in AGEs- and high glucose-treated cells.

### Elevated Bcl-2 levels may be responsible for the impaired adipogenesis in AGEs- and high glucose-treated 3T3-L1 cells

**Figure 4:**
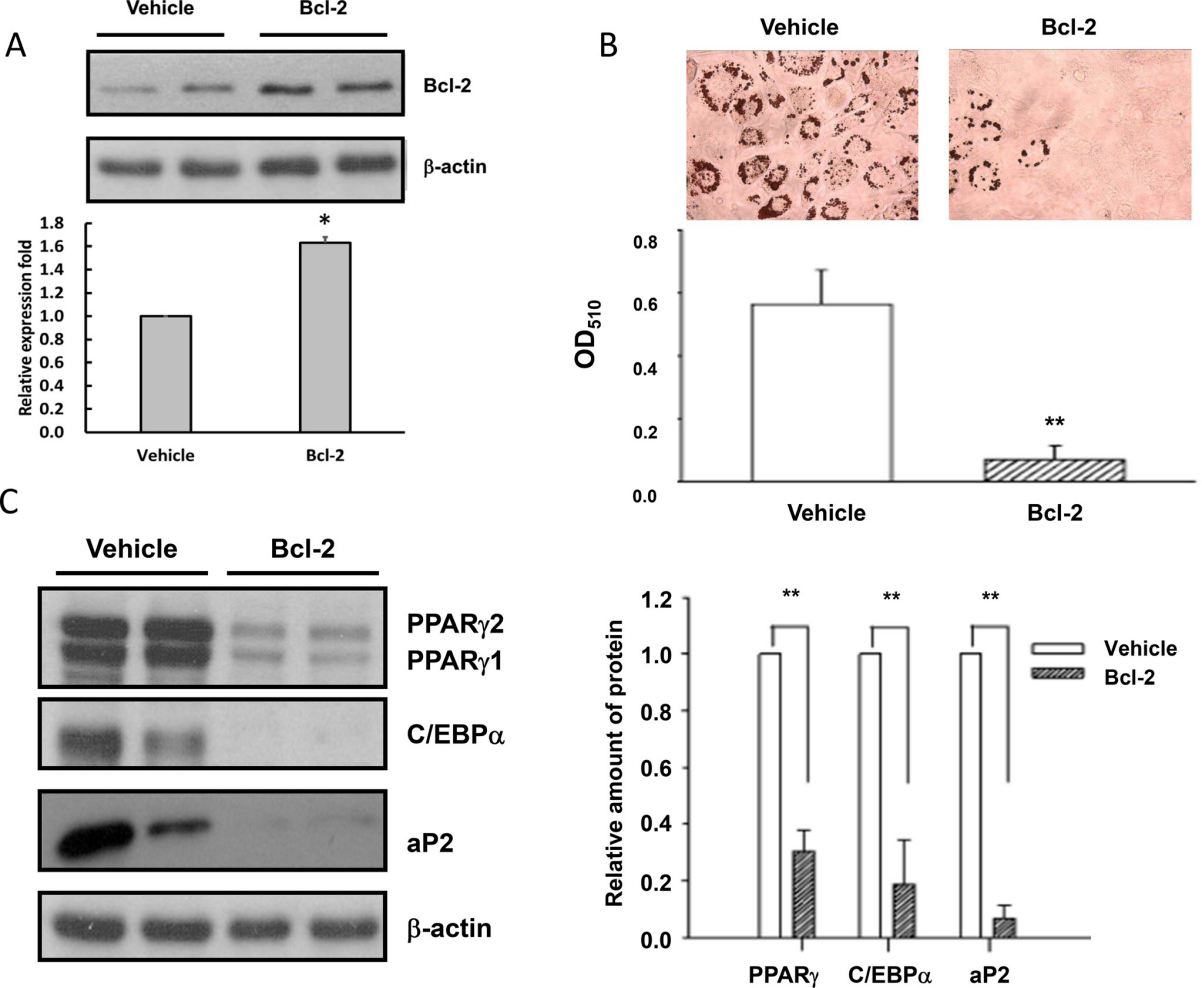
Elevated Bcl-2 levels are responsible for the impaired adipogenesis in AGEs- and high glucose-treated 3T3-L1 cells Cell lysates from 3T3-L1-Vehicle and 3T3-L1-Bcl-2 cells were subjected to Western blotting with Bcl-2 (**A**), PPARγ, C/EBPα, aP2 (**C**) or β-actin antibodies. (**B**) 3T3-L1-Vehicle and 3T3-L1-Bcl-2 cells were induced to differentiate into adipocytes for 5 days and accumulated lipid droplets were visualized by the Oil red O staining. Representative microscope images and quantitation were shown. Bands were quantified by densitometry analysis, and values shown are the means ± SEM of three independent experiments. *p < 0.05 and **p < 0.01, as compared to Vehicle control group, respectively.

To examine whether elevated Bcl-2 levels lead to inhibition of adipogenesis, we generated a stable 3T3-L1 cell line over-expressing Bcl-2 (3T3-L1-Bcl-2 cells) (Figure 4A), and compared the differentiating ability of 3T3-L1-Bcl-2 cells with that of control cells (3T3-L1-Vehicle cells). The results showed that the differentiation of 3T3-L1-Bcl-2 cells was impaired as evidenced by reduced ORO staining extents of oil droplets (Figure 4B) in 3T3-L1-Bcl-2 cells as compared to those in 3T3-L1-Vehicle cells. Similarly, PPARγ C/EBPα and aP2 levels on day 5 after inducing differentiation in 3T3-L1-Bcl-2 cells were much lower than those in 3T3-L1-Vehicle cells (Figure 4C). These results support the hypothesis that elevated Bcl-2 expression is at least partly responsible for the inhibition of adipogenesis by chronic AGEs and high glucose treatments.

### Chronic AGEs and high glucose treatments up-regulate YAP via Akt and NF-κB

**Figure 5:**
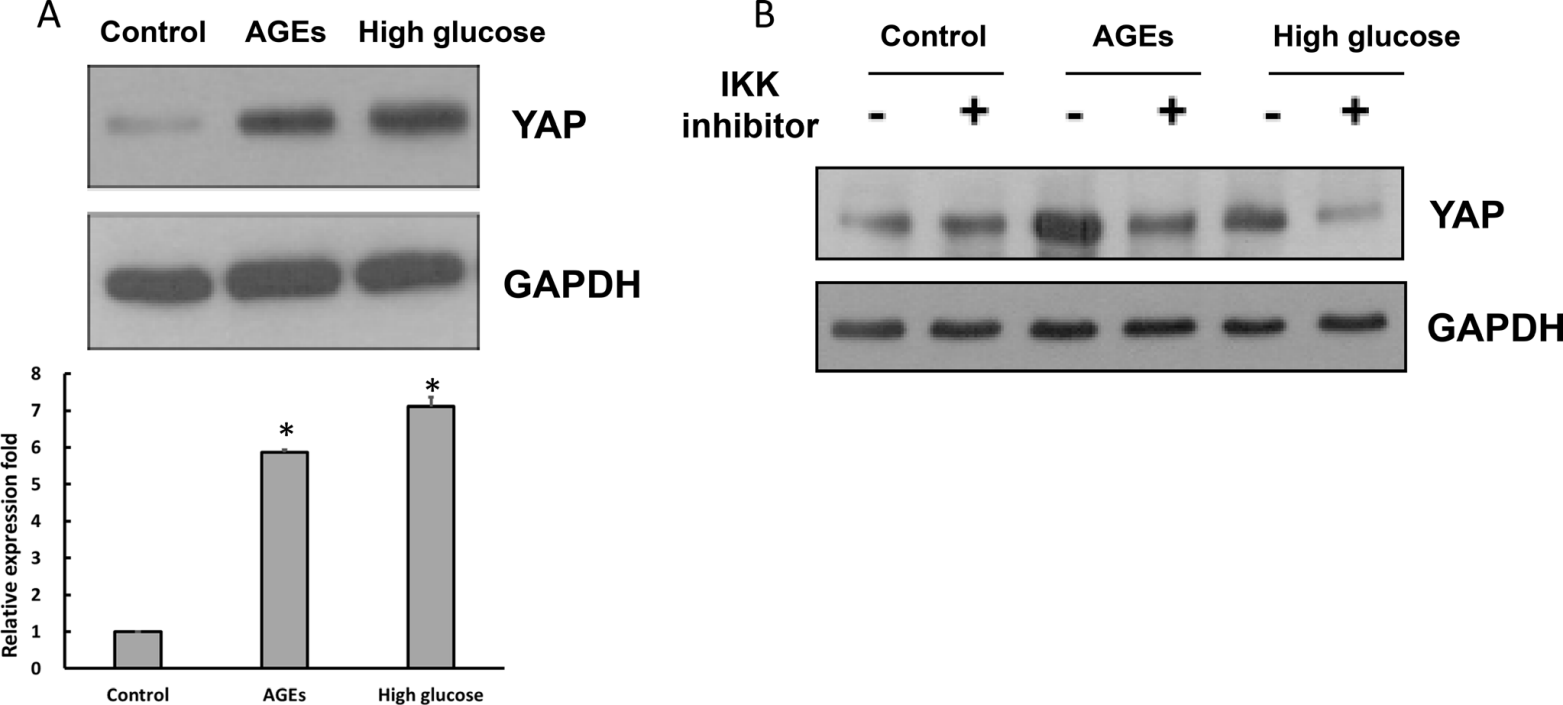
The elevated YAP expression levels in high glucose- and AGEs-treated 3T3-L1 cells (**A**) Control, AGEs- and 25 mM glucose-treated cells lysates were subjected to Western blot analysis using YAP or GAPDH antibodies. (**B**) With or without 1 μM IKK inhibitor 48-hour treatment, cell lysates were harvested and subjected to Western blot analysis using YAP or GAPDH antibodies. Bands were quantified by densitometry analysis, and relative values are listed as the means ± SEM of three independent experiments. *p < 0.05, as compared to control group.

YAP, an effector protein of the Hippo pathway, is known to regulate cell proliferation, apoptosis and differentiation [29–31]. We compared levels of YAP in control, AGEs- and high glucose-treated 3T3-L1 cells, and found that YAP levels were elevated substantially in AGEs- and high glucose-treated 3T3-L1 cells (Figure 5A). Addition of IKK inhibitor attenuated YAP levels in AGEs- and high glucose-treated 3T3-L1 cells (Figure 5B), suggesting that YAP is up-regulated by elevated NF-κB activation in AGEs- and high glucose-treated 3T3-L1 cells.

**Figure 6:**
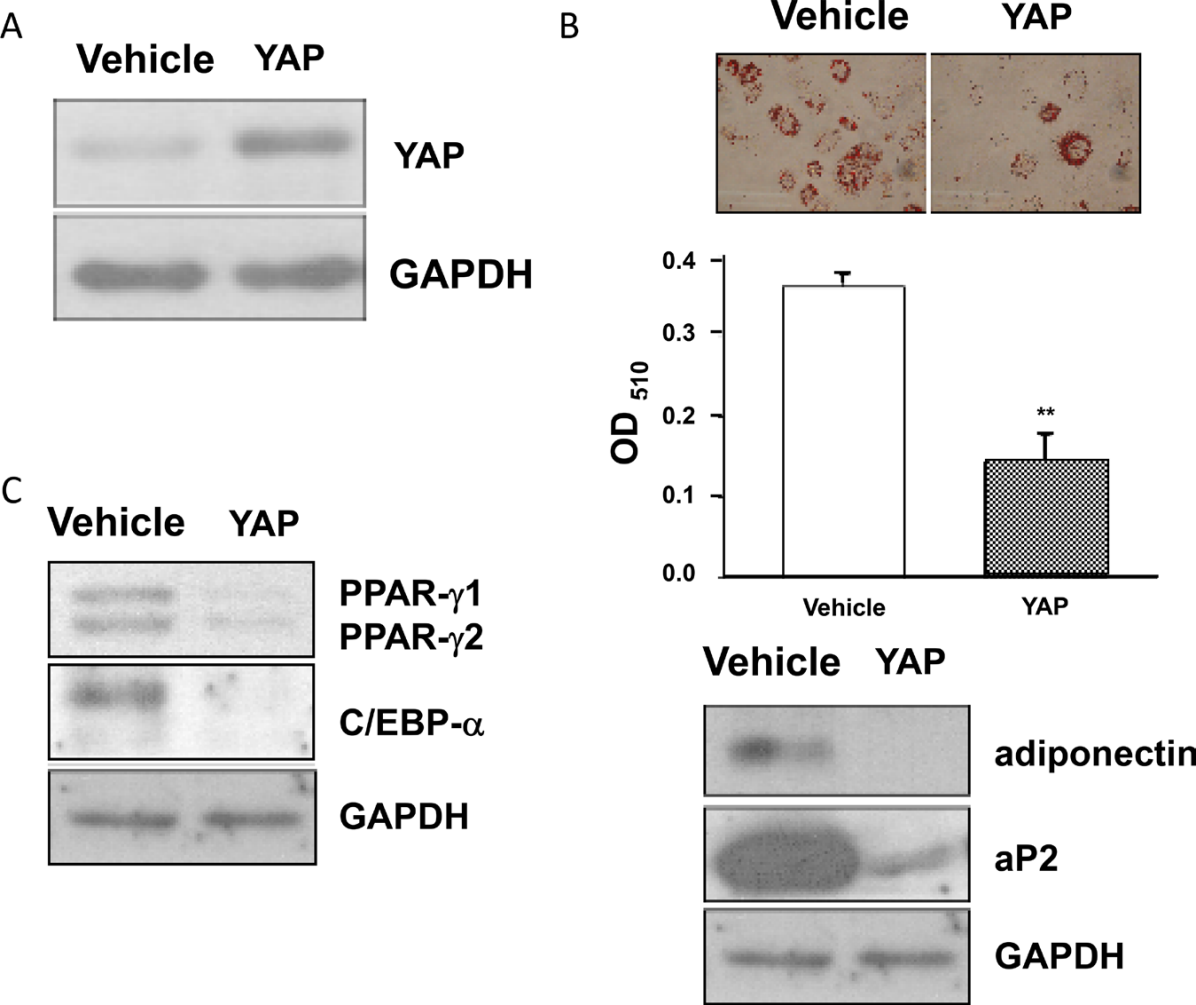
The blockage of adipogenesis in YAP overexpressed 3T3-L1 cells Stable 3T3-L1-YAP and 3T3-L1-Vehicle transfected preadipocytes were harvested and their cell lysates were subjected to Western blot analysis using YAP (**A**), PPARγ, C/EBPα, adiponectin, aP2 (**B**), or GAPDH antibodies. The representative results of three independent experiments were shown in the figures. *(*p* < 0.05) indicates the statistical significant difference between YAP and Vehicle groups.

To determine whether elevated YAP levels affect adipogenesis, we generated cells that stably expressed YAP (3T3-L1-YAP cells) (Figure 6A). Induction of the adipogenic differentiation of 3T3-L1-YAP and 3T3-L1-Vehicle cells revealed that the differentiation of 3T3-L1-YAP cells was substantially attenuated as evidenced by reduced levels of adipocyte markers such as accumulated lipid droplets, adiponectin and aP2 (Figure 6B), and adipogenic transcription factors such as PPARγ and C/EBPα (Figure 6C). These results indicated that elevated YAP levels induced by AGEs and high glucose treatments contribute to the inhibition of adipogenesis.

## DISCUSSION

Chronic hyperglycemia which is characteristics of aging and diabetes facilitates the formation of AGEs [4–7]. Since adipocytes are slow turnover cells [19], adipose tissues in aged or diabetic subjects are exposed to high glucose or AGEs for a prolonged period. However, effects of long-term exposure to AGEs and hyperglycemia on the differentiation of preadipocytes remain unclear. In this study, we investigated the effects of 1 month exposure of 3T3-L1 preadipocytes to AGEs or 25 mM glucose on adipogenesis, and found that prolonged exposure to AGEs and 25 mM glucose inhibited the differentiation of 3T3-L1 preadipocytes.

Our results showed that chronic treatment of 3T3-L1 cells with AGEs or 25 mM glucose blocked the adipogenic differentiation of treated cells as judged by the lack of lipid droplet accumulation and the reduced expression of adipocyte markers such as aP2 and adiponectin. C/EBPα and PPARγ are key transcriptional factors regulating adipogenesis [32–36]. We found that the expression levels of PPARγ and C/EBPα were much lower in AGEs- and 25 mM glucose-treated cells as compared to those in 3T3-L1 cells. The failure of AGEs- and 25 mM glucose-treated cells to differentiate into mature adipocytes may be due to reduced PPARγ and C/EBPα levels.

We further explored the mechanism by which AGEs and 25 mM glucose inhibit adipogenesis. Our results indicated that Src tyrosine kinase activity was elevated in AGEs- and high glucose-treated 3T3-L1 cells. Src has been shown to activate the PI3-kinase-PDK1-Akt pathway [21–23]. Our data showed that inhibition of Src with PP2 attenuated Akt, but not PDK-1, activity in AGEs- and high glucose-treated 3T3-L1 cells, suggesting that Src is upstream of Akt, but not PI3-kinase nor PDK1. On the other hand, since PDK1 protein levels and PDK1 phosphorylation were elevated in AGEs- and high glucose-treated 3T3-L1 cells, the results suggest that PI3-kinase and PDK1 are activated in AGEs- and high glucose-treated 3T3-L1 cells. Thus, chronic AGEs and 25 mM glucose treatments activate Akt via the Src and PI3-kinase-PDK1 pathways.

Akt is known to up-regulate Bcl-2 by the activation of NF-κB [26–28]. Our data revealed that IκB levels were reduced, whereas nuclear NF-κB levels were higher in AGEs- and high glucose-treated cells than those in 3T3-L1 cells, suggesting that NF-κB is constitutively activated in AGEs- and high glucose-treated cells. Moreover, levels of Bcl-2 were elevated in AGEs- and high glucose-treated cells. Up-regulation of Bcl-2 was attenuated by inhibitors of PI3-kinase, Akt and IKKα in AGEs- and high glucose-treated cells, suggesting that elevated Akt activity results in activation of NF-κB lead to up-regulation of Bcl-2 in AGEs- and high glucose-treated cells.

To examine whether elevated Bcl-2 levels contribute to the inhibition of adipogenesis in AGEs- and high glucose-treated cells, stable 3T3-L1 cell lines over-expressing Bcl-2 (3T3-L1-Bcl-2 cells) were generated. Intriguingly, like AGEs- and high glucose-treated cells, the differentiation of 3T3-L1-Bcl-2 cells was impaired as evidenced by the reduced amount of accumulated oil droplets and reduced levels of PPARγ and C/EBPα in 3T3-L1-Bcl-2 cells as compared to 3T3-L1-Vehicle cells. These results suggest that elevated Bcl-2 levels at least partly account for the impaired adipogenesis in AGEs- and high glucose-treated cells. Thus, elevation of the Src-Akt and PI3-kinase-PDK1-Akt pathways leads to activation of NF-κB, up-regulation of Bcl-2, and suppression of adipogenic differentiation in AGEs- and glucose-treated cells. Chronic AGEs and high glucose treatments also up-regulated YAP, another NF-kB-regulated gene.

Like Bcl-2, stable expression of YAP in 3T3-L1 cells attenuated adipogenesis by reducing levels of adipocyte markers such as accumulated oil droplets, adiponectin and aP2 and levels of adipogenic transcription factors C/EBPα and PPARγ. TAZ or WWTR1, the structurally related protein of YAP, has been shown involved in adipogenesis [29–31]. Similarly, our data showed that increasing levels of YAP in 3T3-L1 preadipocytes inhibited the differentiation of 3T3-L1 preadipocytes. Although YAP and TAZ are both effector proteins of the Hippo pathway, they may exert similar or different functions. For instance, both YAP and TAZ serve as transcriptional co-activator for transcription factor partners TEAD and Runx and increase cell proliferation, migration and invasion. However, YAP specifically associates with transcription factors, such as ErbB4, p73, whereas TAZ interacts with PPARγ, Pax3, TBX5, and TTF-1. Moreover, gene knockout mouse studies revealed that knockout of the YAP gene in mice leads to early developmental arrest. However, TAZ knockout mice are viable. In the case of adipogenesis, both YAP and TAZ inhibit adipogenic differentiation. Since YAP levels in AGEs- and high glucose-treated 3T3-L1 cells were attenuated by an IKK inhibitor, suggesting that YAP is a NF-κB downstream gene. Thus, elevated NF-κB activity induced by chronic AGEs and high glucose treatments inhibits adipogenesis via Bcl-2 and YAP.

It is documented that the ability of preadipocytes to differentiate into mature adipocytes declines with age. Expression of C/EBPα, C/EBPδ, and PPARγ declines substantially with aging in preadipocytes from rats of various ages [1–3]. However, the mechanisms by which aging affects these adipogenic transcription factors and inhibits adipogenesis remain unclear. Aging is characterized by mild hyperglycemia and AGEs accumulation which is known to cause cell senescence. Lowering the content of AGEs in the normal diet has been recently shown to significantly prevent AGEs accumulation and to extend lifespan in mice [37]. Thus, hyperglycemia and AGEs may mimic some of the effects of aging. Similar to aging, we found that the expression of C/EBPα and PPARγ in AGEs- and 25 mM glucose-treated cells was less than that in control 3T3-L1 cells. Like aging, chronic AGEs- or high glucose-treatment inhibits the differentiation of preadipocytes. Nuclear NF-κB activity has been shown to be up-regulated with age in mouse and rat tissues [38] such as mouse cardiac muscle [39], rat brain [40], lymphoid organs [41] and gastric mucosa [42]. The NF-κB motif has also been shown to be strongly associated with age in gene expression levels of nine tissue types in mice and humans [43]. Our study also showed that NF-κB activity was activated and its target genes Bcl-2 and YAP were elevated in AGEs- and high glucose-treated 3T3-L1 cells. Therefore, AGEs and hyperglycemia may mediate at least some of the effects of aging via the activation of NF-kB and elevation of Bcl-2 and YAP leading to the inhibition of adipogenesis.

In summary, we have found that prolonged AGEs and high glucose treatments inhibited the differentiation of 3T3-L1 preadipocytes into adipocytes. Our data implied that the Src-Akt and PI3-kinase-PDK1-Akt pathways were activated in AGEs- and high glucose-treated 3T3-L1 cells. Akt subsequently activated NF-κB and up-regulated Bcl-2 and YAP leading to attenuation of the adipogenic differentiation of 3T3-L1 cells.

## MATERIALS AND METHODS

### Reagents and chemicals

LY294002 and PP2 were obtained from Tocris (Bristol, UK). IKK inhibitor VII was purchased from Merck Millipore (Lake Placid, NY, USA). Dulbecco's modified Eagle medium (DMEM) with high and low glucose contents were purchased from ThermoFisher Scientific (Waltham, MA, USA). Fetal bovine serum (FBS), Lipofectamine 2000 transfection reagent and the TRIzol Reagent were purchased from Life Technologies (Grand Island, NY, USA). The enhanced chemiluminescence blotting detection system was obtained from Perkin Elmer (Waltham, MA, USA). Antibodies against phospho-PDK1 (S^241^) and β-actin were purchased from BD Bioscience (San Jose, CA, USA). Antibodies against PPARγ, C/EBPβ, C/EBPδ, aP2, PDK1, phospho-Src (Y^416^), Src, Akt, phospho-Akt (S^473^), phospho-Akt (T^308^), Bcl-2, NF-κB, and IκB were purchased from Cell Signaling (Danvers, MA, USA). Anti-YAP antibody was purchased from Novus Biologicals (Littleton, CA, USA). Antibodies against PTEN and phospho-tyrosine, PureProteome magnetic beads and the Compartmental Protein Extraction kit were purchased from Millipore (Billerica, MA, USA). Antibodies against GAPDH, lamin B1 and C/EBPα were purchased from Santa Cruz Biotechnology (Santa Cruz, CA, USA). The RevertAid H Minus First Strand cDNA Synthesis Kit was purchased from Fermentas (Glen Burnie, MD, USA). Akt Inhibitor VIII trifluoroacetate salt hydrate and other common chemicals were obtained from Sigma (St. Louis, MO, USA).

### Preparation of advanced glycated end product (AGEs)

AGEs were prepared as described previously by incubating bovine serum albumin (10 mg/mL) with 33 mM glyceraldehyde and 100 U/mL penicillin/streptomycin in 0.02 M sodium phosphate buffer (pH 7.4) at 37°C for 3 days in the dark. Generated AGEs were subjected to dialysis against 0.02 M phosphate buffer (pH 7.4) at 4°C for 48 hours and then sterilized by passing through a 0.22 μm filter [18]. No endotoxin was detected in AGEs preparations using endotoxin assay kit from Genscript (Piscataway, NJ, USA).

### Differentiation of 3T3-L1 preadipocytes

3T3-L1 preadipocytes (from Bioresource Collection and Research Center, Food Industry Research and Development Institute, Hsinchu, Taiwan) were maintained in DMEM containing 10% FBS, 3.7 g /L NaHCO_3_, 25 mM HEPES, 1 g/L glucose and 100 U/mL penicillin/streptomycin at 37°C, 5% CO_2_ and 95% humidity. Two days after confluence, preadipocytes were induced differentiation by incubating cells with DMEM containing 0.5 mM IBMX, 1 μM dexamethasone, 1.7 μM insulin, and 10% FBS at 37°C in a 5% CO_2_ humidified incubator for 72 hours. On day 3, the medium was replaced with fresh medium containing 1.7 μM insulin. After day 5, the culture medium was replaced every two days [18, 20].

### Oil red O staining

3T3-L1 adipocytes were rinsed with PBS twice and fixed with 4% paraformaldehyde at room temperature for 1 hour. After fixing, cells were washed with PBS and incubated with a 60°C pre-warmed 0.2% Oil Red O solution at 37°C for 1 hour. Cells were washed with PBS and lipid droplets were visualized and quantified by measuring the absorbance at 510 nm [18, 20].

### Glycerol-3-phosphate dehydrogenase (GPDH) assay

GPDH activities were performed as previously described [18, 44]. Briefly, differentiated adipocyte lysates were extracted with the homogenization buffer containing 0.25 M sucrose, 1 mM EDTA, 1 mM dithiothreitol, and 5 mM Tris-HCl (pH 7.6) on day 8 after adipogenic induction. Homogenates were sonicated and centrifuged (13,400 × g) for 10 min at 4°C. Protein amounts in supernatants were determined with the BCA assay and the enzyme activities (U/μg protein/minute) were obtained from the decline of absorbance of NADH at 340 nm.

### Generation of a Bcl-2 stable cell line

3T3-L1 preadipocytes were transfected by Lipofectamine 2000 with 2.5 μg of plasmid construct carrying mouse Bcl-2 cDNA in pcDNA3.1 (Life Technologies, Carlsbad, CA, USA) or pcDNA3.1 (as the Vehicle control). The following day, cells were then split and pcDNA3.1-Bcl-2 and pcDNA3.1 stable cell lines were selected by culture medium containing 500 μg/mL G418 [18].

### Immunoprecipitation and immunoblotting

For total cell lysate preparation, cells were lysed with RIPA-B lysis buffer (150 mM NaCl, 200 mM Na_2_HPO_4_, 1% Triton X-100, 0.1 M NaF, 2 mM Na_3_VO_4_, 1 mM PMSF, 0.8 μM aprotinin, and 20 μM leupeptin). Extraction of nuclear proteins was conducted with the Compartmental Protein Extraction kit. The PureProteome magnetic beads were used to pull down target proteins from 500 μg total proteins. Twenty five micrograms of total proteins were subjected to SDS-PAGE, and then transferred to the Immobilon-P PVDF membrane. Detection of specific proteins was performed with the enhanced chemiluminescence blotting detection system [18, 44, 45].

### Total RNA extraction and reverse transcriptase polymerase chain reaction (RT-PCR)

Total RNAs were extracted using the TRIzol Reagent. Three micrograms of total RNAs were used to generate cDNA using the RevertAid H Minus First Strand cDNA Synthesis Kit. The cDNA primer sequences for aP2 were 5′-TCTCACCTGGAAGACAGCTCCTCCTCG-3′ (forward) and 5′-TTCCATCCAGGCCTCTTCCTT TGGCTC-3′ (reverse), for adiponectin were 5′-TGATGGC AGAGATGGCACTC-3′ (forward) and 5′-TTCTCCAGG CTCTCCTTTCC-3′ (reverse), and for the internal control 18S were 5′-GGGAGCCTGAGAAACGGC-3′ (forward) and 5′-CCGCTCCCAAGATCCAACTAC-3′ (reverse). PCR amplification was carried out as follows: initial denaturation (95°C, 2 min); 30 and 25 cycles of 95°C 30 s, 56°C 30 s, 72°C 30 s for aP2 and 18S genes, respectively; a final extension step was 72°C for 5 min. For adiponectin gene amplification, a ‘stepdown’ thermal procedure was used: an initial 10 cycles of 94°C 30 s, 74 to 54 °C 15 s (−2°C per cycle), 72°C 30 s, and followed by 94°C 30 s, 54°C 15 s, 72°C 30 s for 25 cycles, the final extension step was 72°C for 5 min. The PCR products were analyzed by agarose gel electrophoresis and visualized by ethidium bromide. The relative levels of genes were normalized with 18S expression.

### Statistical analysis

Data were presented as the means ± SEM. Comparisons between experimental groups were performed by using student's *t*-test for two groups or One-way ANOVA for more than two groups. If there are significant difference in ANOVA test, Tukey's method was applied for multiple comparisons. Differences were considered significant as the *p* value was less than 0.05. Symbols * and ** were used to represent the statistical significances for *p* < 0.05 and *p* < 0.01 as compared to control or the indicated group, respectively.

## SUPPLEMENTARY MATERIALS FIGURES



## References

[1] Kirkland JL, Tchkonia T, Pirtskhalava T, Han J, Karagiannides I (2002). Adipogenesis and aging: does aging make fat go MAD?. Exp Gerontol.

[2] Cartwright MJ, Tchkonia T, Kirkland JL (2007). Aging in adipocytes: potential impact of inherent, depot-specific mechanisms. Exp Gerontol.

[3] Huffman DM, Barzilai N (2009). Role of visceral adipose tissue in aging. Biochim Biophys Acta.

[4] Ahmed N, Thornalley PJ (2007). Advanced glycation endproducts: what is their relevance to diabetic complications?. Diabetes Obes Metab.

[5] Nass N, Bartling B, Navarrete Santos A, Scheubel RJ, Borgermann J, Silber RE, Simm A (2007). Advanced glycation end products, diabetes and ageing. Z Gerontol Geriatr.

[6] Grillo MA, Colombatto S (2008). Advanced glycation end-products (AGEs): involvement in aging and in neurodegenerative diseases. Amino Acids.

[7] Bohlender JM, Franke S, Stein G, Wolf G (2005). Advanced glycation end products and the kidney. Am J Physiol Renal Physiol.

[8] Thomas MC, Forbes JM, Cooper ME (2005). Advanced glycation end products and diabetic nephropathy. Am J Ther.

[9] Stitt AW, Curtis TM (2005). Advanced glycation and retinal pathology during diabetes. Pharmacol Rep.

[10] Yan SF, Ramasamy R, Schmidt AM (2009). Receptor for AGE (RAGE) and its ligands-cast into leading roles in diabetes and the inflammatory response. J Mol Med (Berl).

[11] Aguiari P, Leo S, Zavan B, Vindigni V, Rimessi A, Bianchi K, Franzin C, Cortivo R, Rossato M, Vettor R, Abatangelo G, Pozzan T, Pinton P (2008). High glucose induces adipogenic differentiation of muscle-derived stem cells. Proc Natl Acad Sci USA.

[12] Chuang CC, Yang RS, Tsai KS, Ho FM, Liu SH (2007). Hyperglycemia enhances adipogenic induction of lipid accumulation: involvement of extracellular signal-regulated protein kinase 1/2, phosphoinositide 3-kinase/Akt, and peroxisome proliferator-activated receptor gamma signaling. Endocrinology.

[13] Gagnon A, Sorisky A (1998). The effect of glucose concentration on insulin-induced 3T3-L1 adipose cell differentiation. Obes Res.

[14] Lin Y, Berg AH, Iyengar P, Lam TK, Giacca A, Combs TP, Rajala MW, Du X, Rollman B, Li W, Hawkins M, Barzilai N, Rhodes CJ (2005). The hyperglycemia-induced inflammatory response in adipocytes: the role of reactive oxygen species. J Biol Chem.

[15] Kume S, Kato S, Yamagishi S, Inagaki Y, Ueda S, Arima N, Okawa T, Kojiro M, Nagata K (2005). Advanced glycation end-products attenuate human mesenchymal stem cells and prevent cognate differentiation into adipose tissue, cartilage, and bone. J Bone Miner Res.

[16] Unoki H, Bujo H, Yamagishi S, Takeuchi M, Imaizumi T, Saito Y (2007). Advanced glycation end products attenuate cellular insulin sensitivity by increasing the generation of intracellular reactive oxygen species in adipocytes. Diabetes Res Clin Pract.

[17] Wu CH, Huang HW, Huang SM, Lin JA, Yeh CT, Yen GC (2011). AGE-induced interference of glucose uptake and transport as a possible cause of insulin resistance in adipocytes. J Agric Food Chem.

[18] Yang SJ, Chen CY, Chang GD, Wen HC, Chen CY, Chang SC, Liao JF, Chang CH (2013). Activation of Akt by advanced glycation end products (AGEs): involvement of IGF-1 receptor and caveolin-1. PLoS One.

[19] Neese RA, Misell LM, Turner S, Chu A, Kim J, Cesar D, Hoh R, Antelo F, Strawford A, McCune JM, Christiansen M, Hellerstein MK (2002). Measurement *in vivo* of proliferation rates of slow turnover cells by 2H2O labeling of the deoxyribose moiety of DNA. Proc Natl Acad Sci USA.

[20] Wang YF, Zhang CX, Wu H, Zhang A, Wang JC, Zhang SF, Pu JL (2015). Modulation of phase behaviors and charge carrier mobilities by linkage length in discotic liquid crystal dimers. Soft Matter.

[21] Arcaro A, Aubert M, Espinosa del Hierro ME, Khanzada UK, Angelidou S, Tetley TD, Bittermann AG, Frame MC, Seckl MJ (2007). Critical role for lipid raft-associated Src kinases in activation of PI3K-Akt signalling. Cell Signal.

[22] Jin W, Yun C, Hobbie A, Martin MJ, Sorensen PH, Kim SJ (2007). Cellular transformation and activation of the phosphoinositide-3-kinase-Akt cascade by the ETV6-NTRK3 chimeric tyrosine kinase requires c-Src. Cancer Res.

[23] Koga F, Xu W, Karpova TS, McNally JG, Baron R, Neckers L (2006). Hsp90 inhibition transiently activates Src kinase and promotes Src-dependent Akt and Erk activation. Proc Natl Acad Sci USA.

[24] Chen YC, Chen CH, Hsu YH, Chen TH, Sue YM, Cheng CY, Chen TW (2011). Leptin reduces gentamicin-induced apoptosis in rat renal tubular cells via the PI3K-Akt signaling pathway. Eur J Pharmacol.

[25] Jin YP, Fishbein MC, Said JW, Jindra PT, Rajalingam R, Rozengurt E, Reed EF (2004). Anti-HLA class I antibody-mediated activation of the PI3K/Akt signaling pathway and induction of Bcl-2 and Bcl-xL expression in endothelial cells. Hum Immunol.

[26] Tamatani M, Che YH, Matsuzaki H, Ogawa S, Okado H, Miyake S, Mizuno T, Tohyama M (1999). Tumor necrosis factor induces Bcl-2 and Bcl-x expression through NFkappaB activation in primary hippocampal neurons. J Biol Chem.

[27] Kane LP, Shapiro VS, Stokoe D, Weiss A (1999). Induction of NF-kappaB by the Akt/PKB kinase. Curr Biol.

[28] Ozes ON, Mayo LD, Gustin JA, Pfeffer SR, Pfeffer LM, Donner DB (1999). NF-kappaB activation by tumour necrosis factor requires the Akt serine-threonine kinase. Nature.

[29] Dong J, Feldmann G, Huang J, Wu S, Zhang N, Comerford SA, Gayyed MF, Anders RA, Maitra A, Pan D (2007). Elucidation of a universal size-control mechanism in Drosophila and mammals. Cell.

[30] Zhao B, Wei X, Li W, Udan RS, Yang Q, Kim J, Xie J, Ikenoue T, Yu J, Li L, Zheng P, Ye K, Chinnaiyan A (2007). Inactivation of YAP oncoprotein by the Hippo pathway is involved in cell contact inhibition and tissue growth control. Genes Dev.

[31] Zhang H, Pasolli HA, Fuchs E (2011). Yes-associated protein (YAP) transcriptional coactivator functions in balancing growth and differentiation in skin. Proc Natl Acad Sci USA.

[32] Barak Y, Nelson MC, Ong ES, Jones YZ, Ruiz-Lozano P, Chien KR, Koder A, Evans RM (1999). PPAR gamma is required for placental, cardiac, and adipose tissue development. Mol Cell.

[33] Rosen ED, Sarraf P, Troy AE, Bradwin G, Moore K, Milstone DS, Spiegelman BM, Mortensen RM (1999). PPAR gamma is required for the differentiation of adipose tissue *in vivo* and *in vitro*. Mol Cell.

[34] Tontonoz P, Spiegelman BM (2008). Fat and beyond: the diverse biology of PPARgamma. Annu Rev Biochem.

[35] Wang ND, Finegold MJ, Bradley A, Ou CN, Abdelsayed SV, Wilde MD, Taylor LR, Wilson DR, Darlington GJ (1995). Impaired energy homeostasis in C/EBP alpha knockout mice. Science.

[36] Freytag SO, Paielli DL, Gilbert JD (1994). Ectopic expression of the CCAAT/enhancer-binding protein alpha promotes the adipogenic program in a variety of mouse fibroblastic cells. Genes Dev.

[37] Peppa M, Uribarri J, Vlassara H (2008). Aging and glycoxidant stress. Hormones (Athens).

[38] Helenius M, Hanninen M, Lehtinen SK, Salminen A (1996). Aging-induced up-regulation of nuclear binding activities of oxidative stress responsive NF-kB transcription factor in mouse cardiac muscle. J Mol Cell Cardiol.

[39] Helenius M, Hanninen M, Lehtinen SK, Salminen A (1996). Changes associated with aging and replicative senescence in the regulation of transcription factor nuclear factor-kappa B. Biochem J.

[40] Korhonen P, Helenius M, Salminen A (1997). Age-related changes in the regulation of transcription factor NF-kappa B in rat brain. Neurosci Lett.

[41] Spencer NF, Poynter ME, Im SY, Daynes RA (1997). Constitutive activation of NF-kappa B in an animal model of aging. Int Immunol.

[42] Xiao ZQ, Majumdar AP (2000). Induction of transcriptional activity of AP-1 and NF-kappaB in the gastric mucosa during aging. Am J Physiol Gastrointest Liver Physiol.

[43] Adler AS, Sinha S, Kawahara TL, Zhang JY, Segal E, Chang HY (2007). Motif module map reveals enforcement of aging by continual NF-kappaB activity. Genes Dev.

[44] Wise LS, Green H (1979). Participation of one isozyme of cytosolic glycerophosphate dehydrogenase in the adipose conversion of 3T3 cells. J Biol Chem.

[45] Chen ZJ, Vetter M, Chang GD, Liu S, Che D, Ding Y, Kim SS, Chang CH (2004). Cyclophilin A functions as an endogenous inhibitor for membrane-bound guanylate cyclase-A. Hypertension.

