# Supplementary material for: Hyperglycemia and advanced glycation end products (AGEs) suppress the differentiation of 3T3-L1 preadipocytes
Source: Oncotarget. 2017 Jul 5;8(33):55039–50. doi: 10.18632/oncotarget.18993 (PMC5589639; doi:10.18632/oncotarget.18993)
Supplement: Supplementary file 1 [file oncotarget-08-55039-s001.pdf]

# Hyperglycemia and advanced glycation end products (AGEs) suppress the differentiation of 3T3-L1 preadipocytes

## SUPPLEMENTARY MATERIALS

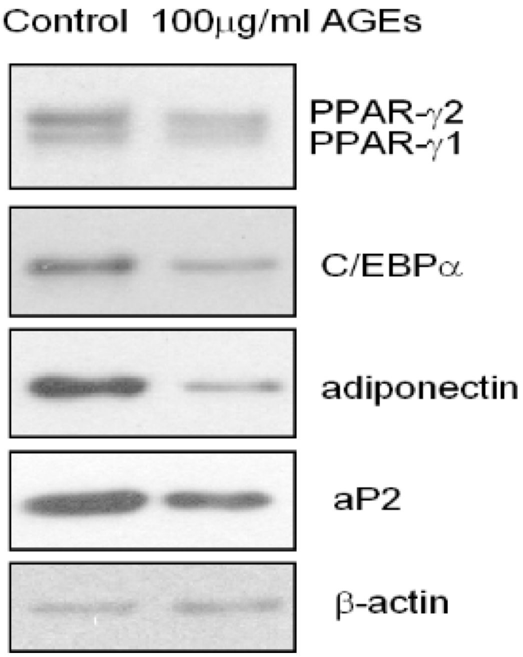


**Supplementary Figure 1: Blockage of 3T3-L1 preadipocyte differentiation by AGEs.** 3T3-L1 and 100 μg/ml AGEs-treated preadipocytes were induced to differentiate into adipocytes. Eight days after induction, cell lysates were collected and immunoblotted with PPARγ, C/EBPα, adiponectin, aP2 and β-actin antibodies.

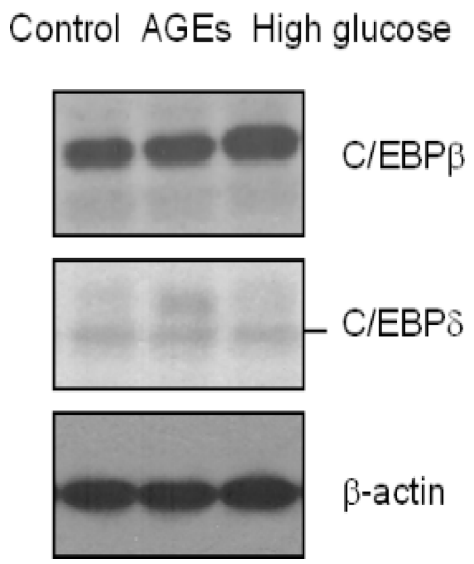


**Supplementary Figure 2: Expression levels of C/EBPβ and C/EBPδ in control, AGEs- and high glucose-treated 3T3-L1 adipocytes.** 3T3-L1, AGEs-treated and 25 mM glucose-treated preadipocytes were differentiated into adipocytes. One day after induction, cell lysates from control, AGEs- and high glucose-treated cells were immunoblotted with C/EBPβ, C/EBPδ and β-actin antibodies.
